# Hepatic arterial spin labelling MRI: an initial evaluation in mice

**DOI:** 10.1002/nbm.3251

**Published:** 2014-12-17

**Authors:** R Ramasawmy, A E Campbell-Washburn, J A Wells, S P Johnson, R B Pedley, S Walker-Samuel, M F Lythgoe

**Affiliations:** a UCL Centre for Advanced Biomedical ImagingPaul O'Gorman Building, London, UK; b UCL Cancer InstitutePaul O'Gorman Building, London, UK

**Keywords:** liver, ASL, perfusion, mouse, repeatability, metastasis, variability, preclinical

## Abstract

The development of strategies to combat hepatic disease and augment tissue regeneration has created a need for methods to assess regional liver function. Liver perfusion imaging has the potential to fulfil this need, across a range of hepatic diseases, alongside the assessment of therapeutic response. In this study, the feasibility of hepatic arterial spin labelling (HASL) was assessed for the first time in mice at 9.4 T, its variability and repeatability were evaluated, and it was applied to a model of colorectal liver metastasis. Data were acquired using flow-sensitive alternating inversion recovery-arterial spin labelling (FAIR-ASL) with a Look–Locker readout, and analysed using retrospective respiratory gating and a *T*_1_-based quantification. This study shows that preclinical HASL is feasible and exhibits good repeatability and reproducibility. Mean estimated liver perfusion was 2.2 ± 0.8 mL/g/min (mean ± standard error, *n* = 10), which agrees well with previous measurements using invasive approaches. Estimates of the variation gave a within-session coefficient of variation (CV_WS_) of 7%, a between-session coefficient of variation (CV_BS_) of 9% and a between-animal coefficient of variation (CV_A_) of 15%. The within-session Bland–Altman repeatability coefficient (RC_WS_) was 18% and the between-session repeatability coefficient (RC_BS_) was 29%. Finally, the HASL method was applied to a mouse model of liver metastasis, in which significantly lower mean perfusion (1.1 ± 0.5 mL/g/min, *n* = 6) was measured within the tumours, as seen by fluorescence histology. These data indicate that precise and accurate liver perfusion estimates can be achieved using ASL techniques, and provide a platform for future studies investigating hepatic perfusion in mouse models of disease. Copyright © 2014 John Wiley & Sons, Ltd.

## Introduction

The application of arterial spin labelling (ASL) to the liver has not been undertaken extensively, possibly because of the liver's complex blood supply and respiratory-induced artefacts, and has been restricted to a limited number of clinical investigations (1–2). Alternative approaches, such as contrast-enhanced computed tomography, Doppler ultrasound and radioactive microspheres (3), have used perfusion to predict the onset of hepatocyte dysfunction (4), monitor tumour therapy (5) and inform on post-transplant success (6). Moreover, MRI assessment of liver disease pathology is well suited to the longitudinal evaluation of disease progression and therapeutic response in experimental models (7). A non-invasive and contrast agent-free method that could robustly measure liver perfusion would benefit researchers investigating a range of liver conditions, including cirrhosis (4) and either primary or metastatic tumours (8).

Currently, dynamic contrast-enhanced (DCE)-MRI is the most common MRI measure of liver perfusion in clinical research, in which the pharmacokinetics of a chelated gadolinium contrast agent are modelled (9). The vasculature of the liver is unique, in that the portal vein delivers approximately 75% of the blood (10), whilst the remaining 25% is drawn from the hepatic artery. This dual supply means that the quantification of liver blood flow can be challenging, and requires careful consideration of acquisition and modelling methods. For dual input quantification, arterial and venous phases in the signal enhancement curve must be separated, which can be challenging in small animal models of disease, as a result of limited temporal resolution (11–12), although recent clinical studies have adopted advanced acquisition strategies to improve this (13). Moreover, the liver is significantly affected by respiratory motion, and the management of the interaction of respiratory gating with the passage of a bolus of contrast agent during arterial and portal phases can be particularly challenging. However, some success has been reported in the measurement of the ratio of the arterial and venous contributions via the hepatic perfusion index, which has been shown to be informative for a number of liver diseases (14).

Conversely, ASL-MRI is not reliant on external contrast agent administration, thereby offering a key advantage over DCE-MRI, and the approach taken in this study was to estimate the total (both portal and arterial) regional delivery to the liver. ASL-MRI has been developed preclinically and has been utilised to measure perfusion in the brain, heart and kidneys (15–17). Despite the potential to fulfil the need for robust and reliable non-invasive liver perfusion measurement, ASL has not yet been reported in the liver of small animals, although a few human studies have been published (1–2), (18–19). Here, we report the feasibility of a flow-sensitive alternating inversion recovery (FAIR) (20) sequence in the liver, which is a form of pulsed ASL that estimates perfusion from two *T*_1_ measurements following slice-selective and global inversion pulses, centred on the imaging slice.

For image acquisition during inversion recovery, a segmented Look–Locker sampling technique was implemented (21), because of the signal-to-noise ratio (SNR) efficiency of the sequence and its previous demonstration for the measurement of myocardial perfusion in mice (22). This gradient-echo acquisition is advantageous over traditional echo planar imaging readouts used in ASL methods because of its reduced sensitivity to image artefacts caused by magnetic susceptibility and motion, particularly in small animal livers. This free-breathing approach is sensitive to image artefacts caused by motion and, although the liver is less clearly affected by motion than the heart, its position below the diaphragm makes it susceptible to respiratory motion. Various strategies have therefore been investigated in this study for the retrospective gating of hepatic ASL (HASL) data. The technique's variability and repeatability are assessed in order to inform future preclinical studies of mouse liver perfusion in models of pathology and the assessment of novel therapies. Finally, HASL is applied to a mouse model of liver metastasis to assess the technique's sensitivity to hepatic disease.

## Materials and Methods

### Animal models

All animal studies were conducted in accordance with the UK Home Office Animals Science Procedures Act (1986). Mice were allowed to feed *ad libitum*. Ten female BALB/C mice, aged 6–8 weeks, were individually anaesthetised with isoflurane (3% in 1 L O_2_/min for induction) and placed in a supine position within the bore of a 9.4-T MRI scanner (approximately 1.9% isoflurane in 1 L O_2_/min during scanning). Subject temperatures were monitored and maintained using heated water pipes, and respiratory bellows were placed below the sternum for physiological monitoring (SA Instruments Inc., Stony Brook, NY, USA).

The mouse model of colorectal liver metastasis was established using an intra-splenic injection of one million SW1222 colorectal tumour cells in six immune-compromised MF1 *nu/nu* mice (23). Imaging was performed at 4 weeks post-implantation, as determined by bioluminescent imaging. Following MRI, mice were intravenously injected (15 mg/kg) with the perfusion marker Hoechst 33342 (Cambridge Bioscience, Cambridge, UK) and blood vessels were stained using the vascular endothelial marker CD31 (Abcam, Cambridge, UK). Fluorescence images were taken on an Axio Imager microscope (Carl Zeiss, Oberkochen, Germany), using an AxioCam digital colour camera.

### Pulse sequences

MRI was performed on a 9.4-T MRI scanner (Agilent Technologies, Santa Clara, CA, USA) with a 39-mm-diameter birdcage coil (RAPID Biomed, Rimpar, Germany). Mice were weighed immediately before being placed into the scanner.

An axial, respiratory-gated, high-resolution, *T*_2_-weighted, multi-slice fast spin echo sequence was used to determine a suitable and consistent slice for HASL measurements. An axial imaging plane was chosen to optimally image both the portal vein and the hepatic artery for anatomical consistency between imaging sessions. Image parameters included: field of view (FOV), 30 × 30 mm^2^; matrix size, 192 × 192; TR = 1500 ms; effective TE = 19 ms; three averages; 0.5-mm-thick slices which covered the liver; imaging time, ≈15 min.

**Figure 1:**
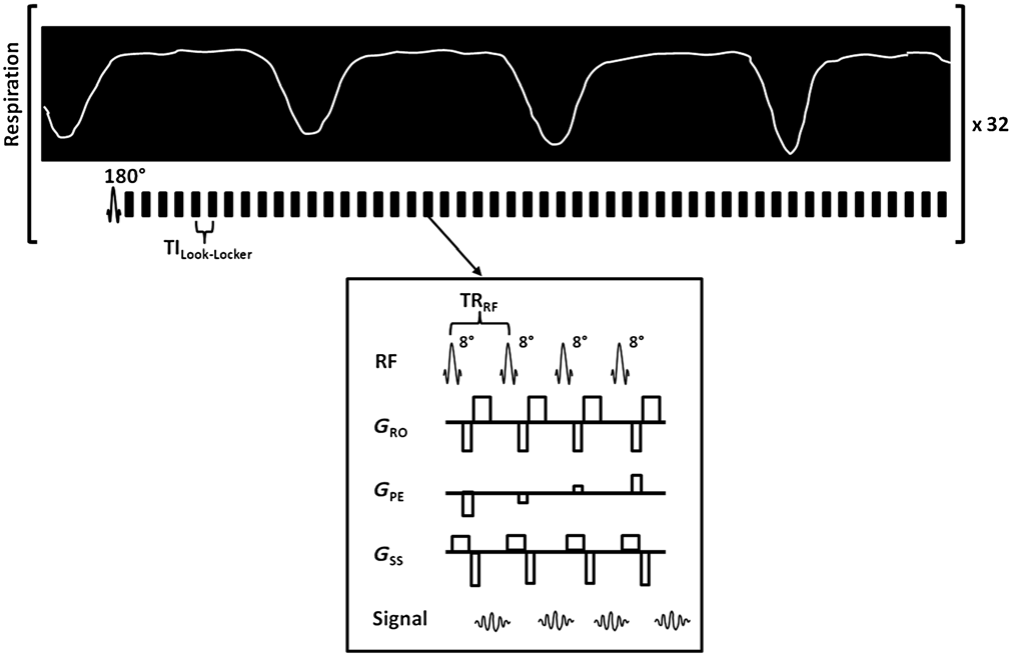
Schematic diagram of the respiratory-triggered, Look–Locker *T*_1_ mapping sequence with a spoiled, single-slice, gradient-echo readout. The inversion pulse is end-expiration triggered, followed by a free-breathing, segmented, Look–Locker sampling train. Each segmented block is separated by TI_Look–Locker_; each block contains four sampling pulses with TR_RF_. The sequence is performed twice with differing inversion slice thicknesses as part of the flow-sensitive alternating inversion recovery-arterial spin labelling (FAIR-ASL) design; a hepatic ASL (HASL) dataset is completed in 15 min.

A single-slice, segmented, Look–Locker FAIR sequence with a gradient-echo readout was used to acquire HASL data (Fig.1); the inversion pulse was respiratory triggered and the following Look–Locker acquisition was free breathing. Sequence parameters included: 1-mm-thick slices; FOV, 25 × 25 mm^2^; matrix size, 128 × 128; TE = 1.18 ms; inversion time spacing (TI_Look–Locker_), 110 ms; repetition time between Look–Locker sampling pulses (TR_RF_), 2.3 ms; sampling flip angle (α_RF_), 8°; TR_I_ = 13 s; 50 inversion recovery readouts; four lines per segmented acquisition with 50 sampling points. A 6-mm localised selective inversion was followed by a global inversion, with the scan taking around 15 min. An adiabatic fast-passage inversion pulse with a length of 2 ms and bandwidth of 20 kHz was used for slice-selective and global preparations, and 500-µs Gaussian radiofrequency pulses were used for Look–Locker excitation.

### *T*_1_ and perfusion estimates

Total liver perfusion (*P*) maps were quantified pixel-wise using the Belle model (16), which has been developed and demonstrated previously for measurements in the mouse myocardium (Equation [1]) (22). A *T*_1_-based perfusion quantification following a FAIR-ASL preparation provides an alternative method to measure total liver blood flow to overcome the quantification challenges of hepatic perfusion. Any blood flowing into the slice will cause an apparent acceleration of the longitudinal relaxation, and the Look–Locker readout provides an efficient method to accurately characterise the tissue relaxation. Perfusion estimates from this model will thus include both arterial and portal contributions, given by the difference in slice-selective (*T*_1, selective_) and global (*T*_1, global_) longitudinal relaxation measurements, assuming a constant blood inflow.


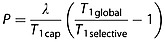
(1)

A blood–tissue partition coefficient *λ* = 0.95 mL/g was taken from previously reported ^85^Kr gas clearance measurements (24). *T*_1,cap_ is the intravascular capillary blood longitudinal relaxation time, which has been measured in the ventricular blood pool in the mouse heart to have a value of 1900 ms at 9.4 T (22). High (non-physiological) perfusion deriving from the major blood vessels was removed by automatically thresholding the perfusion values above 20 mL/g/min (16), and noise-induced negative perfusion values were set to zero. Prior to pixel-wise *T*_1_ fitting, an edge-preserving filter (25) was applied to all data. *T*_1_ maps were generated in MATLAB (MathWorks, Natick, MA, USA) using a non-linear least-squares algorithm.

### Look–Locker saturation correction

In order to sample the longitudinal magnetisation during its recovery following an inversion pulse, the Look–Locker sequence applies a train of small flip angle readout pulses, separated by a fixed TI. This pulse train partially saturates the tissue signal, resulting in an apparent longitudinal relaxation time *T*_1_* that is shorter than the true *T*_1_ time. A three-parameter fit can be applied to the saturated inversion recovery using Equation [2]. Assuming a perfect inversion, the true *T*_1_ can be estimated (Equation [3]) (26) using the saturated inversion efficiency (*β*) from Equation [2]:


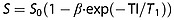
(2)


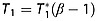
(3)

### Data conditioning

The free-breathing Look–Locker *T*_1_ measurement resulted in some lines of *k* space being acquired during inspiration and expiration phases. In-house analysis software was written in MATLAB to detect images in the ASL dataset that were severely corrupted by respiratory motion. Three retrospective data conditioning modes were compared: (i) no conditioning; (ii) image rejection as a result of the acquisition of the central three lines of *k* space during a breath as recorded by a digital data-logger (Cambridge Electronic Design, Cambridge, UK) (22); (iii) image rejection using the phase-encoded noise-based image rejection (PENIR) algorithm.

Previous mouse myocardium ASL measurements used a method of retrospective gating of the Look–Locker data using physiology recordings as acquired using a digital data-logger. A method rejecting images from *T*_1_ quantification as a result of the central lines of *k* space being acquired during respiratory motion was reported (mode ii), and is applied in this study.

**Figure 2:**
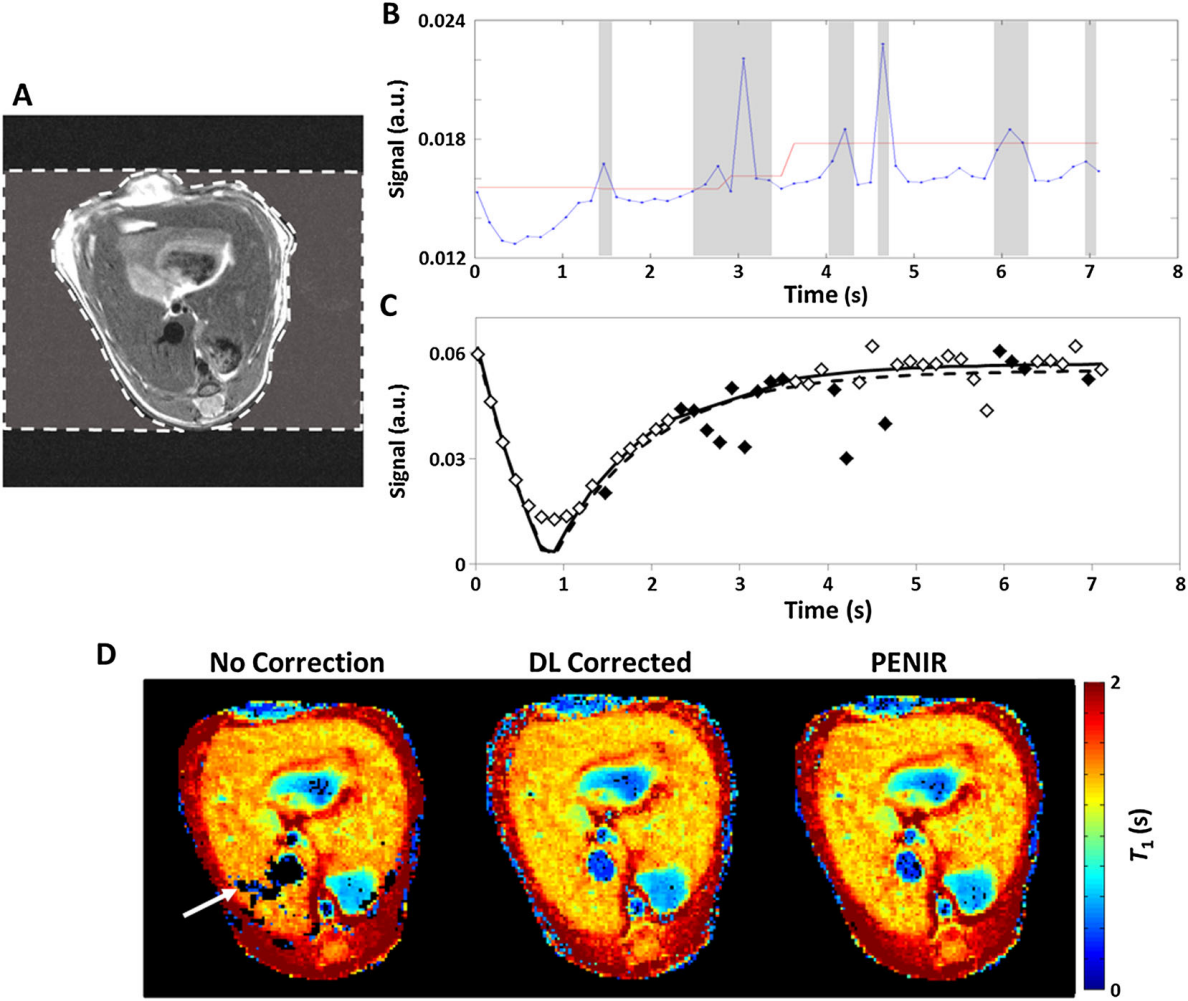
The phase-encoded noise-based image rejection (PENIR) scheme for retrospective removal of motion-corrupted data. (A) Axial image of a mouse liver with an example user-determined region of interest (ROI) (broken line) of the extracorporeal area in the phase-encoding direction. (B) Significant increases in the mean noise signal (blue line) above a signal-generated threshold (red line) will result in image omission (shaded areas). (C) Inversion recovery fitting in a liver region will typically overestimate *T*_1_ without correction (broken line) compared with rejection (full line) because of reduced tissue signal from corrupted images (filled diamonds). (D) A comparison of *T*_1_ maps shows that, with no correction, the respiration artefacts may lead to unsuccessful fitting and generate areas of signal dropout (arrow). With both data-logger (DL) and PENIR retrospective gating, these areas of dropout are recovered – no significant differences in mean *T*_1_ were measured between these two retrospective conditioning modes (*t*-test, *p* > 0.05).

The PENIR scheme was initially developed in-house for retrospective gating where respiratory trace recordings for Look–Locker FAIR perfusion imaging were not available. PENIR exploits the presence of ‘ghost’ artefacts in the phase-encoding direction (Fig.2). PENIR highlights corrupted images by observing spikes in a user-determined region of interest (ROI) from the extracorporeal space (Fig.2A). From this area, the mean signal (Fig.2B, blue line) will be expected to spike as a result of respiratory motion-induced ghosting. A threshold was generated from the mean noise signal plus one standard deviation in the remaining extracorporeal field and was calculated every 10 TIs. When the spikes significantly exceeded the data-generated threshold (red line), images acquired at these inversion times were attributed to respiratory-induced artefacts and rejected from *T*_1_ fitting. Typically, the signal within a liver parenchyma ROI is reduced as a result of motion artefacts (filled diamonds, Fig.2C), which leads to an overestimation of *T*_1_ with a concomitant effect on perfusion estimates. A Tukey analysis of variance (ANOVA) was performed between the data conditioning rejection criteria in SPSS (IBM, Armonk, NY, USA).

### Repeatability and reproducibility assessment

For the repeatability and reproducibility study, 10 female Balb/C mice were scanned under the same protocol, 1 week apart. Each protocol included a high-resolution, respiratory-gated fast spin echo sequence, which was used to determine the location of the porta hepatis, where both the hepatic artery and portal vein are visible. Four consecutive HASL scans were then performed, with the animal maintained at a constant level of anaesthesia and temperature.

### Statistical analysis

The coefficient of variation (CV) (Equation [4]), a normalised measure of the data variability given by the ratio of the standard deviation *σ* to the mean *μ*, was used to compare measurements of liver perfusion:


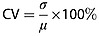
(4)

Three variability measures were calculated. Repeated measurements within an imaging session were used to assess the within-session CV (CV_WS_ ± standard error). The between-session CV (CV_BS_) was estimated from a comparison of index-matched scans between week 1 and week 2 for each mouse (i.e. week 1-scan 1 with week 2-scan 1). The between-animal variation (CV_A_), reflecting the variability between perfusion estimates in different animals, was calculated from all ASL scans. Each coefficient was calculated from a region encompassing all the liver parenchyma within the slice. The inter-animal precision was obtained from the standard deviation of perfusion estimates between animals.

To determine the minimum detectable change in perfusion given by hepatic ASL, the Bland–Altman repeatability coefficient (RC) (Equation [5]) was calculated. Bland–Altman RCs correspond to the 95% confidence interval given by two perfusion estimates. Δ*P* is the difference in perfusion estimates between time points,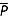 is the mean perfusion and *n* is the sample size:


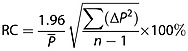
(5)

RCs were calculated from the first two scans within each session, for each week, and averaged to produce the within-session RC_WS_. The between-session RC_BS_ was generated from averaging the RCs of number-matched scans between the weeks for all the animals.

## Results

### Hepatic arterial spin labelling

**Figure 3:**
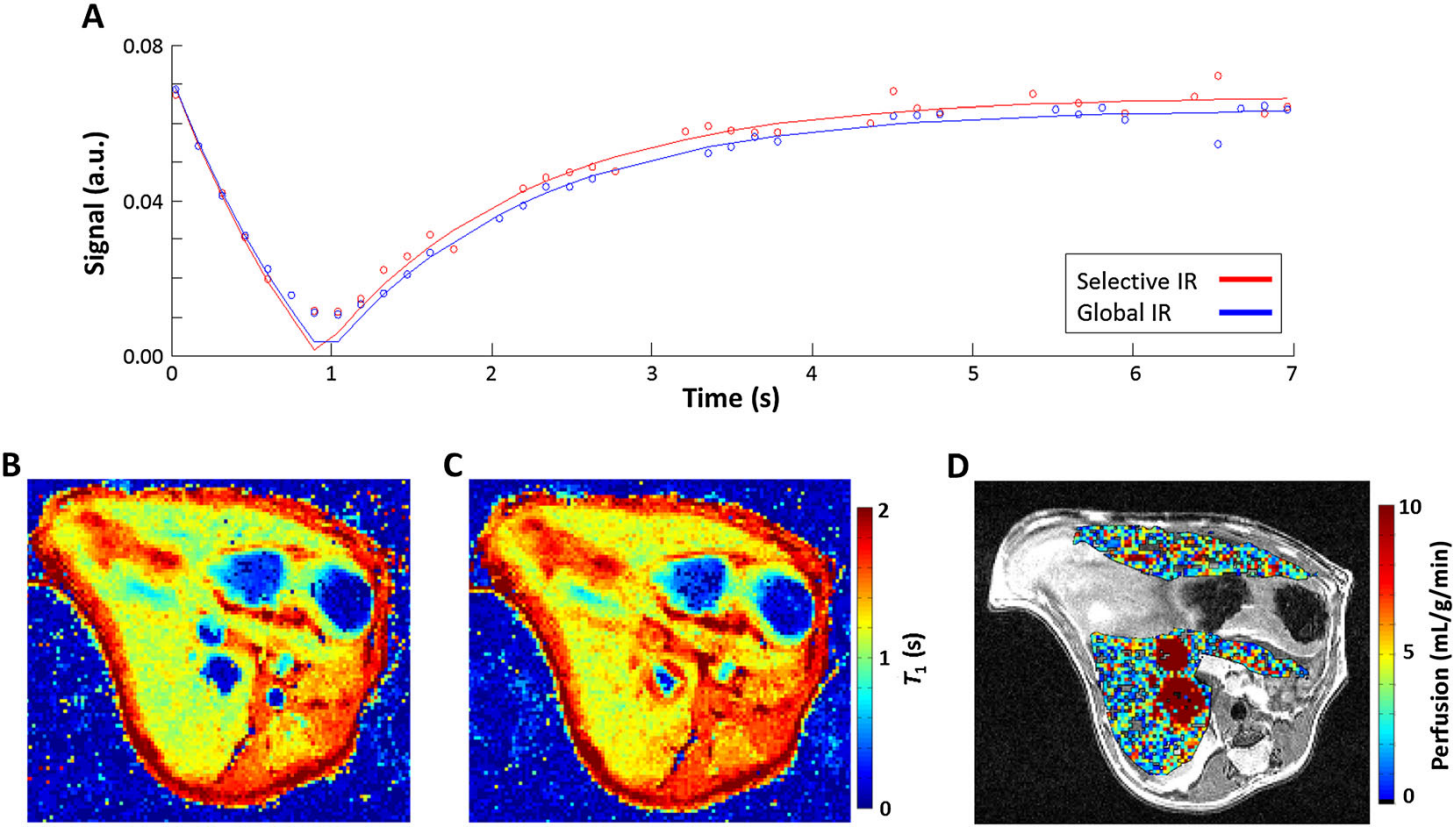
Quantification of perfusion from a hepatic arterial spin labelling (HASL) dataset. (A) The perfusion signal comes from the difference in longitudinal recovery following slice-selective (red) and global (blue) inversion. Example *T*_1_ maps acquired after a slice-selective inversion (B) and global inversion (C); *T*_1_ values have been clipped at 2 s (mean liver global *T*_1_ = 1.36 ± 0.06 s). (D) In the resulting perfusion map, the liver parenchyma has been overlaid on a high-resolution *T*_2_-weighted fast spin echo image. Non-physiologically high perfusion values can be seen within major blood vessels; perfusion values in this image have been limited at 10 mL/g/min. Mean estimated liver perfusion was 2.2 ± 0.8 mL/g/min (mean ± standard error, *n* = 10).

Example images from the quantification of perfusion from HASL data are shown in Figure3. The mean signal within a liver tissue ROI illustrates the difference in longitudinal relaxation rates that are observed following slice-selective (red) and global (blue) inversion (Fig.3A). Here, respiration-corrupted images have been removed from the data. Example *T*_1_ maps from pixel-wise fitting of the data for slice-selective and global inversions are shown in Figure3B, C, respectively. From these images, mean global *T*_1_ measured in the liver was 1.36 ± 0.06 s. Fast spin echo images with the perfusion map overlaid on the liver parenchyma, such as those shown in Figure3D, revealed that high, non-physiological perfusion values are present within the major vasculature, as the perfusion model is not valid in these regions. Mean liver perfusion, estimated using Equation [1] in all datasets acquired in this study, was 2.2 ± 0.3 mL/g/min (mean ± standard deviation). The blood vessels were excluded from the perfusion estimate by an ROI drawn on the slice-selective *T*_1_ maps and, additionally, by removing perfusion values above 20 mL/g/min.

### Assessment of data conditioning modes

The differences in retrospective gating on *T*_1_ mapping can be visualised in Figure2D: the dataset with no conditioning [mode (i)] exhibits areas of signal dropout (arrow) which correspond to data that cannot successfully be fitted to the inversion profile as a result of motion artefact-induced signal deviations. The *T*_1_ maps following gating using the data-logger [DL, mode (ii)] and PENIR [mode (iii)] successfully recover these areas, and do not show any significant differences between them.

Using data conditioning mode (ii), in which images with motion-corrupted central lines of *k* space were rejected using the data-logger, only 34% of the 50 readout images remained following rejection, whereas the PENIR scheme [mode (iii)] maintained approximately 54% of images. Data from nine of the 10 datasets were evaluated for mode (ii), as one set of data was unusable because of a malfunction in respiratory trace recording. No significant difference in perfusion estimates was measured between modes (ii) and (iii). Mode (i), no rejection, gave perfusion estimates that were non-significantly higher (*p* > 0.2, Tukey ANOVA) than the respiratory-corrected estimates (on average by 19%), which is consistent with the results of a previous study (22). Comparing modes (ii) and (iii), the data-logger method would occasionally discard data that did not display visually obvious artefacts because of an overly wide user-defined gating window employed in the physiological monitoring software. The PENIR scheme produced a smaller uncertainty in the least-squares three-parameter fit, compared with mode (i) and (ii) results. Therefore, in all further experiments, mode (iii) was used for data conditioning.

### Repeatability and reproducibility of liver perfusion estimates

A significant increase in animal mass was measured from week 1 (18.5 ± 0.6 g) to week 2 (18.9 ± 0.4 g) (*p* = 0.02, paired *t*-test). No significant difference in respiratory rate during scanning was observed between weeks 1 and 2 (49 ± 8 breaths/min in week 1 and 49 ± 9 breaths/min in week 2, *p* > 0.37). Furthermore, no significant correlation was measured between estimated liver perfusion and animal weight, breathing rate or isoflurane concentration, which was individually maintained at a constant level, but varied from 1.7 to 2.2% (flow rate, 1 L O_2_/min) across the subjects. No influence of anaesthesia was expected, as previous measurements did not find a significant change in total liver blood flow over these relatively small isoflurane concentration variations (27).

**Figure 4:**
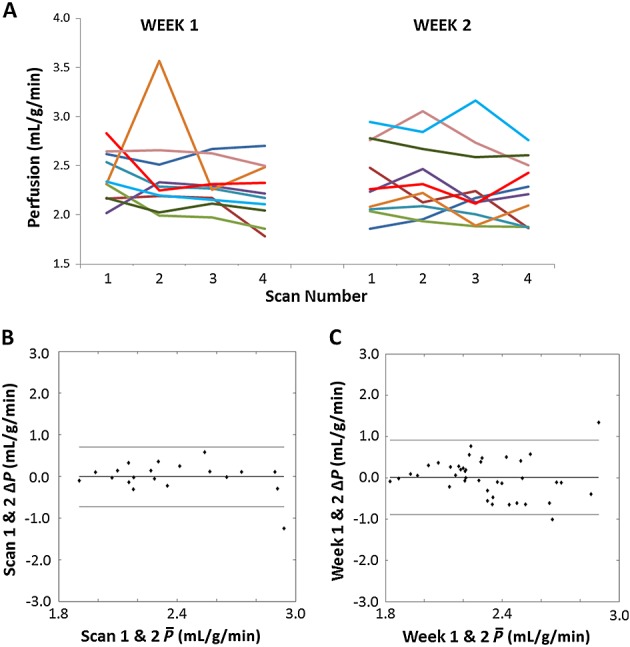
(A) The variability in mean liver perfusion across all 10 animals (shown in different colours). The second imaging session was 1 week later. Across the group, no significant trends were observed within and between the imaging sessions. Bland–Altman plots showing the within-session repeatability from the first and second mean liver perfusion estimate (B) and the between-session mean perfusion repeatability from index-matched scans (C). The central, thick full line represents the mean difference, and the two outer lines show the ±1.96 × standard deviation. For both plots, the mean difference measured was within the error of the technique, and no trends could be distinguished.

The variation within each perfusion imaging session across the cohort can be visualised in Figure4A, and the mean session perfusion estimates for all animals and both weeks are shown in Table 1. Normalising each session to the first perfusion estimate, no significant trends in perfusion were measured during the session duration. There was no significant difference between the first and last perfusion estimates across all the imaging sessions (paired *t*-test, *p* > 0.2). In addition, there was no between-session perfusion trends observed when comparing scan index-matched perfusion estimates between weeks 1 and 2 (paired *t*-test, *p* > 0.4).

**Table 1:** Variability of liver perfusion estimates (session average ± standard deviation in mL/g/min) across the 10 mice for both weeks

	Subject									

Week	1	2	3	4	5	6	7	8	9	10
1	2.6 ± 0.1	2.1 ± 0.2	2.0 ± 0.2	2.2 ± 0.1	2.3 ± 0.2	2.7 ± 0.6	2.4 ± 0.3	2.6 ± 0.1	2.1 ± 0.1	2.2 ± 0.1
2	2.1 ± 0.2	2.2 ± 0.3	1.9 ± 0.1	2.3 ± 0.1	2.0 ± 0.1	2.1 ± 0.1	2.3 ± 0.1	2.8 ± 0.2	2.7 ± 0.1	2.9 ± 0.2

### Coefficient of variation

The CV_WS_ value was 7 ± 1%, CV_BS_ was 9 ± 1% and CV_A_ was 15 ± 1%. The three data conditioning modes investigated produced CVs which differed within 1%. The mean within-session standard deviation was calculated to be ±0.4 mL/g/min.

### Repeatability coefficients

A Bland–Altman plot showing the within-session differences, by comparing perfusion estimates from repeated measurements averaged over the liver, is shown in Figure4B. The plot is not suggestive of magnitude dependence in perfusion estimates and has a mean difference of 0.01 ± 0.34 mL/g/min. Between-session Bland–Altman analysis (Fig.4C) is also free from any magnitude dependence and measured a mean difference of 0.01 ± 0.46 mL/g/min. The Bland–Altman RC provides an estimate of the percentage change required to measure a significant variation in a particular parameter estimate; RC_WS_ was 18% and RC_BS_ was 29%.

### Application to a model of liver metastasis

**Figure 5:**
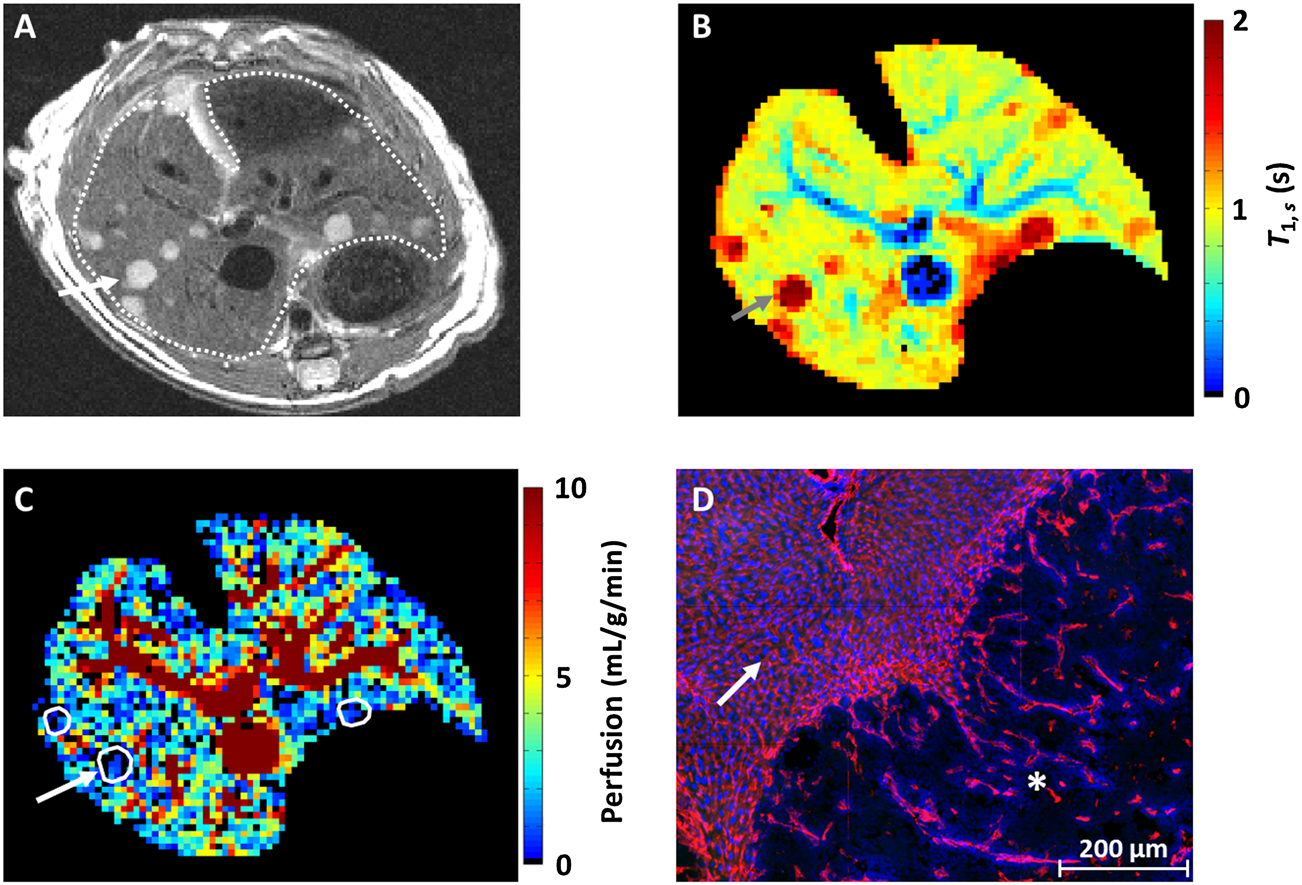
Application of hepatic arterial spin labelling (HASL) to a model of liver metastasis. (A) High-resolution *T*_2_-weighted fast spin echo images show the tumours (arrow) as hyperintense relative to the liver tissue (outlined). (B) In the corresponding slice-selective *T*_1_ map, the metastases can be delineated by a raised *T*_1_ (2.24 ± 0.54 s) relative to liver tissue. (C) Across *n* = 6 mice, a significant reduction in perfusion (outlined) was measured within the metastases (1.1 ± 0.5 mL/g/min, mean ± standard deviation) relative to the liver tissue. This difference in perfusion measured by HASL was confirmed by histology. (D) The fluorescence image shows a section of normal liver containing sinusoid vessels (arrow), demarcated from an adjacent SW1222 colorectal liver metastasis (star) by a large reduction in the presence of blood vessels. Vascular structures are shown in red (anti-CD31 antibody) and perfusion in blue (by the injected marker Hoechst 33342).

Finally, the HASL technique was applied to a model of colorectal liver metastasis (Fig.5). A *T*_2_-weighted fast spin echo image (Fig.5A) facilitated tumour detection, which appeared hyperintense (example, arrow) relative to liver tissue (outlined). Furthermore, *T*_1_ maps produced from HASL data also distinguished tumours from surrounding liver (arrow) because of their longer relaxation times (Fig.5B). Segmentation of tumours was undertaken using *T*_1_ maps estimated from slice-selective inversions, as these provided optimal contrast between liver, tumour tissue and vasculature. Across six animals, a significantly lower perfusion was measured in tumours (1.1 ± 0.5 mL/g/min, mean ± standard deviation) relative to liver tissue (2.4 ± 0.6 mL/g/min, *p* < 0.01, paired *t*-test). This 46% perfusion difference is greater than the measured within-session variability of HASL, and can be observed within the tumours outlined in the perfusion map shown in Figure5C. This perfusion deficit in a well-vascularised model of colorectal liver metastasis is in agreement with fluorescence histology (Fig.5D). The normal-appearing liver sinusoids are well vascularised and perfused (arrow), as shown by the presence of CD31 staining (red) and Hoechst 33342 (blue). The SW1222 colorectal tumour exhibits a reduced number of blood vessels, and thus a reduced amount of perfusion.

## Discussion and Conclusion

We have applied Look–Locker FAIR-ASL-MRI for the first time to measure liver perfusion in a small animal setting. We investigated the reproducibility and repeatability of the technique, within and between imaging sessions. In addition, a method of retrospective respiratory gating (PENIR) was introduced that is data driven, and was found to reduce the uncertainty in the perfusion estimate. Finally, HASL was applied to a mouse model of colorectal liver metastasis and showed a perfusion deficit within the tumours relative to the normal liver.

The mean liver perfusion measured in this study with HASL was 2.2 ± 0.3 mL/g/min, which is in good agreement with previous estimates in the literature. Microsphere measurements in similar weight mice gave a mean total hepatic perfusion of 1.8 ± 0.3 mL/g/min (3). The same study also reported a reference perfusion in the kidneys of 5.1 ± 0.8 mL/g/min, which agrees well with the mean perfusion measured incidentally in the renal cortex by HASL (5.3 ± 0.6 mL/g/min). In addition, a previous study of liver perfusion in mice using DCE-MRI gave a value of 2.48 mL/g/min [reported as 4.96 ± 1.87 mL/min (28), converted to bulk perfusion assuming a mouse liver mass of 2 g (29)].

The majority of previous measurements of liver perfusion using invasive techniques, which have been undertaken in rat rather than mouse liver, report mean liver perfusions of 2.41 ± 0.50 mL/g/min (^85^Kr clearance) (24), 1.9 ± 0.1 mL/g/min (microspheres) (30), 2.03 ± 0.13 mL/g/min (indocyanine green clearance) and 2.28 ± 0.49 mL/g/min (galactose clearance) (31). However, at least one study has compared renal perfusion measured with ASL in both rats and mice (17), and has found that between-species perfusion measurements did not differ significantly. This could conceivably also be reflected in liver perfusion between species.

From the repeatability and reproducibility analysis undertaken in this study, the largest variability was found in measurements between animals, suggesting that subject variability was greater than that of the technique. Global liver perfusion could be a useful quantity when applied to models of cirrhosis and liver dysfunction, in which marked changes are likely to occur (4). In addition, parenchymal *T*_1_ has been reported to vary as a result of fibrosis development (32) and cirrhosis onset (33), which is provided inherently by the Look–Locker FAIR-ASL acquisition.

Isoflurane level has been reported previously to have a significant influence on myocardial perfusion measurements (34), although no such association was found in this study, which is in agreement with previous liver studies (27). A further potential source of variability was the feeding status between animals, as animals were allowed to feed *ad libitum*. The portal vein draws blood from the intestines, and so the between-animal variability observed may be exacerbated by differing dietary habits between the mice. Previous behavioural studies in mice have reported a cyclic consumption of food approximately every 24 h (35), however in this study, no trend in perfusion was correlated with the time of day to suggest that an *ad libitum* diet may not be an influential factor to the variability in liver perfusion. Future studies may investigate HASL's sensitivity to post-prandial liver perfusion changes following a controlled diet (19).

Our approach also allowed us to measure much lower perfusion values in a mouse model of liver metastasis, which provides good evidence for the application of HASL to liver pathology. A limitation of the tumour perfusion quantification is that it utilised the liver's blood–tissue partition coefficient; however, previous ASL methods assumed a *λ*_T_ value of unity (36). However, this will only induce a 5% difference in the perfusion estimate, and therefore would not affect the significant differences measured between metastases and liver tissue. A measurable level of perfusion within these neoplasms will facilitate perfusion-based determination of the efficacy of vascular targeting therapy (23).

A limitation of this technique is that the readout was single slice. To provide greater coverage of the liver, the pulse sequence proposed here could be adapted to acquire multiple slices without impacting the total imaging time, but would require quantification methods that account for slice-variant inflow effects (37). The implemented FAIR-ASL preparation and *T*_1_-based quantification are attractive because of their estimation of the total liver blood flow (i.e. both portal venous and arterial) independent from modelled parameters, such as the arterial transit time. However, the perfusion contributions of the hepatic artery and portal vein may be separated using a pseudo-continuous ASL (38) preparation which can distinguish the blood contributions by independently positioning the tagging plane on the descending aorta and portal vein (19). The ratio of arterial to venous perfusion, the hepatic perfusion index, has been used to stage liver tumours (39). However, such a technique is not straightforward to implement in mice because of their small anatomy, and the tagging efficiency may be reduced as a result of respiratory and cardiac motion.

The intrinsic low-perfusion SNR of ASL experiments leads to the majority of methods employing an efficient readout, such as echo planar imaging. However, such techniques can be particularly challenging in the mouse liver as the surrounding abdominal fat and air cavities in the stomach and lungs can make imaging prone to distortion and signal dropout. Hence, a gradient-echo acquisition was used here and a Look–Locker readout was applied to increase the efficiency of the HASL dataset. Although the low sampling flip angle used in a Look–Locker scheme can result in low-SNR images, the large number of TIs acquired improves the *T*_1_ estimation, but still may lead to noise within the perfusion maps. In an alternative set-up, the SNR may be improved using a surface receiver coil, although we found a suboptimal coverage of the liver using such an arrangement.

Currently, DCE-MRI is used more extensively than ASL in the assessment of both liver and tumour perfusion. However, an advantage of ASL over DCE-MRI is the ability to acquire data longitudinally without the need to wait for a contrast agent to clear, which would be particularly advantageous when assessing physiological changes across short timescales (such as assessing the acute response to treatment). Equally, removing the need to perform cannulation of a vein in small animals would be a key advantage of ASL over DCE-MRI. However, a comparison of the relative accuracy and precision associated with each approach would need to be performed.

HASL has good potential for clinical translation, and preliminary evaluation in humans has returned encouraging results (2), (18). As such, this methodology offers great promise in the diagnosis and assessment of response to therapy in a number of hepatic diseases, such as cirrhosis and metastasis, in both the preclinical and clinical environments.

## Abbreviations

*ANOVA*: *analysis of variance*
*ASL*: *arterial spin labelling*
*CV*: *coefficient of variation*
*DCE*: *dynamic contrast enhanced*
*FAIR*: *flow-sensitive alternating inversion recovery*
*FOV*: *field of view*
*HASL*: *hepatic arterial spin labelling*
*PENIR*: *phase-encoded noise-based image rejection*
*RC*: *repeatability coefficient*
*ROI*: *region of interest*
*SNR*: *signal-to-noise ratio.*
